# A comparison of coffee floral traits under two different agricultural practices

**DOI:** 10.1038/s41598-019-43753-y

**Published:** 2019-05-14

**Authors:** Sara Guiti Prado, Jaime A. Collazo, Philip C. Stevenson, Rebecca E. Irwin

**Affiliations:** 1 North Carolina Cooperative Fish and Wildlife Research Unit, Department of Applied Ecology, North Carolina State University, Raleigh, NC 27695 USA; 2 U.S. Geological Survey, North Carolina Cooperative Fish and Wildlife Research Unit, Department of Applied Ecology, North Carolina State University, Raleigh, North Carolina 27695 USA; 3 Royal Botanic Gardens, Kew, Richmond, Surrey, TW9 3AB UK; 4 Natural Resources Institute, University of Greenwich, Chatham Maritime, Kent ME4 4TB UK; 5 Department of Applied Ecology, North Carolina State University, Raleigh, NC 27695 USA

**Keywords:** Environmental impact, Tropical ecology

## Abstract

Floral traits and rewards are important in mediating interactions between plants and pollinators. Agricultural management practices can affect abiotic factors known to influence floral traits; however, our understanding of the links between agricultural practices and floral trait expression is still poorly understood. Variation in floral morphological, nectar, and pollen traits of two important agricultural species, *Coffea arabica* and *C. canephora*, was assessed under different agricultural practices (sun and shade). Corolla diameter and corolla tube length were larger and pollen total nitrogen content greater in shade plantations of *C. canephora* than sun plantations. Corolla tube length and anther filament length were larger in shade plantations of *C. arabica*. No effect of agricultural practice was found on nectar volume, sugar or caffeine concentrations, or pollen production. Pollen total nitrogen content was lower in sun than shade plantations of *C. canephora*, but no difference was found between sun and shade for *C. arabica*. This study provides baseline data on the influence of agronomic practices on *C. arabica* and *C. canephora* floral traits and also helps fill a gap in knowledge about the effects of shade trees on floral traits, which can be pertinent to other agroforestry systems.

## Introduction

Pollination is a critical ecosystem service, with up to 90% of flowering plants requiring insects or other animals for pollination^1^ and approximately 35% of the global plant-based food supply being dependent on animal-mediated pollination^2^. Floral traits and rewards, including nectar and pollen, are important in mediating interactions between plants and pollinators. Pollinators can use a combination of visual and olfactory signals from flowers to determine which patches, plants, and individual flowers to visit^1^. Floral morphology, including anther and stigma heights, can affect how effective different pollinator species are at removing pollen from anthers and depositing it on stigmas^3,4^. Despite the importance of floral traits in pollinator attraction and pollination and well-known examples of pollinator-mediated selection on floral traits^5,6^, there are a surprising number of plant species, including both wild and agricultural species, for which we have little information about variation in their floral morphology and reward chemistry, what influences this and how it affects pollinator visitation and pollination. Floral traits in horticultural crops have been influenced through breeding practices and domestication with potential consequences for pollinators^7–9^, but there is less evidence of how cultivation practices influence floral traits. The goal of this study was therefore to assess variation in morphological and chemical traits of flowers, nectar, and pollen of two important agricultural species, *Coffea arabica* and *C. canephora*, under different farm management strategies.

Floral traits can vary in response to environmental pressures^10,11^. For example, the application of low concentrations of nitrogen-based fertilizer can result in plants with larger flowers, which produce more nectar than plants exposed to higher concentrations of nitrogen^12^. This in turn can result in increased pollinator visitation rates to the low-nitrogen plants^12^. In a similar vein, the shading of flowering species can also affect floral traits and rewards. For example, increased solar irradiance can have a positive effect on nectar production rate of *Thymus capitatus*^13^. Moreover, *Campanulastrum americanum* plants in the sun have larger floral displays and receive seven times more pollinator visits than plants in the shade^14^. While natural variation in nutrient and light availability can affect floral traits important for pollinator visitation and seed production, agricultural management practices can also affect these abiotic factors, which could affect the links between agricultural management, floral trait expression, and pollination. For example, although pumpkin plants may benefit from increased nitrogen inputs by producing larger, more numerous flowers, which produce nectar that is more frequently and abundantly consumed by bumble bees, the bees in turn experience drastically (22%) reduced survival rates after consuming this more attractive nectar^15^.

In coffee production, two primary management strategies are used: growing coffee under shade trees or in full sun. Not only does the amount of sun reaching the coffee plants differ in these two management strategies, but also the amount and timing of nutrient inputs. In shade management, nutrient inputs from fallen leaf litter from shade trees can exceed those of inorganic fertilizers applied in sun management, even when the latter is applied at the highest recommended level for coffee^16^. Moreover, the speed of nutrient release differs between the two management strategies, where the leaf litter allows for a slow and steady release of nutrients in shade management compared to some chemical fertilizers applied in sun management^16,17^. Leaf litter can also retain soil moisture and provide erosion control^18^. Although several studies have assessed the effects of shade vs. sun management on the physiology and production of coffee plants^19–21^, the effects on the expression of floral traits and rewards important for pollination are relatively unknown but may be an important consideration for crops that are dependent on pollinators.

Floral chemistry is also important for pollinator attraction and visitation^22–24^. Secondary metabolites in leaf tissue typically thought to function to deter herbivores are also found in floral rewards, including nectar and pollen^25–27^. Although in certain instances nectar and pollen secondary metabolites can be toxic to pollinators^27–29^, in most cases their effects on pollinators are concentration-dependent (e.g., see refs^30,31^). Effects of nectar secondary metabolites can range from deterrence of, to neutral effects on pollinator visitation^32^, and in some cases can result in positive effects on pollinator visitation^33^. For example, two recent laboratory studies have shown that the alkaloid caffeine found in coffee nectar can enhance pollinator learning and memory of reward^23^, resulting in optimized pollen receipt^22^, with potential benefits for plant reproductive success. However, above 0.1 M, nectar caffeine may act as a deterrent and may even be lethal to bees^30^. Of the two commercially produced coffee species, *C. canephora* is more likely to contain higher concentrations of caffeine in its nectar than *C. arabica*^23^. Although there are potential concentration-dependent benefits of nectar caffeine on coffee pollination, how sun vs. shade management of coffee affects nectar caffeine content is unknown. A study on the effects of shading on caffeine concentration of *C. arabica* bean characteristics showed that coffee beans in shaded plantations have higher caffeine concentrations than those in full sun^34^. As alkaloid concentrations in plants can be positively correlated between different plant parts^35,36^, it is possible that caffeine concentration in coffee flowers will also be higher in shade plantations.

*Coffea arabica* originated almost 50,000 years ago from a natural hybridization between *C. canephora* and *C. eugenioides*^37^. The plant and the leaves of *C. canephora* are generally larger in size than those of *C. arabica*, standing 3–6.5 meters tall, whereas *C. arabica* are usually only measuring up to 5 m^38,39^. However, there is no information on their floral traits, pollen production, protein content, nectar volume and its sugar and caffeine content. These traits, which can affect bee pollinator preferences and visitation rates^40,41^, may vary with coffee cultivation practices. However, the ways in which these may vary is unknown^17^. We compared floral morphology and nectar and pollen quantities and chemistries between sun and shade coffee plantations of *C. arabica* and *C. canephora*, in Puerto Rico. In the absence of specific morphometric data, we first conducted a contrast among flower morphological traits, and then combined all morphometric data by species to assess if there were species-specific floral patterns or patterns between cultivation practices (sun vs. shade). We predicted that flowers under sun would be more exposed to environmental stresses such as soil and atmospheric water deficits, high temperatures, or their combined effects^19,42^, and thus, might be smaller for both species than in shade plantations. If the flowers are indeed smaller, then we would also expect them to contain less nectar and pollen^43^. Alternatively, if coffee plants in full sun are not water deficient, and stomatal aperture is not limited, then they may have higher photosynthetic rates than shaded trees, resulting in increased energy for growth and reproduction^19^. In this case, we would expect flowers of sun plantations to be larger. Additionally, based on prior studies of caffeine content of coffee beans^44,45^, we predicted that flowers of *C. canephora* and shade plantations would have higher nectar caffeine concentrations than those of *C. arabica*, and sun plantations, respectively. We discuss the potential implications of the floral trait differences we observed for pollination success, as well as the conservation and economic implications of our results for shade coffee in Puerto Rico and other regions where alternatives to sun coffee cultivation are being considered.

## Results

### Floral shape

**Figure 1:**
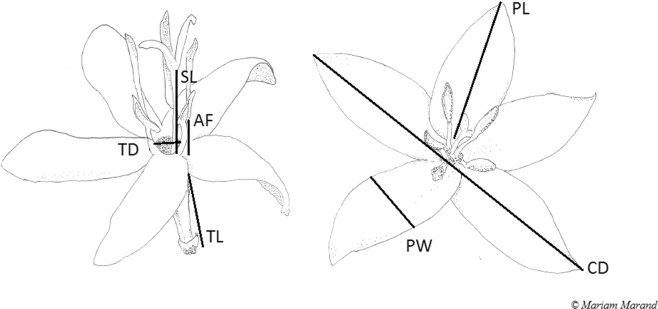
Schematic representation of *Coffea* flowers. Measured floral traits were TD = tube diameter, SL = style length, AF = anther filament length, TL = tube length, PW = petal width, PL = petal length, and CD = corolla diameter. Drawing by Mariam Marand.

We found that many of the floral morphological traits (Fig. 1) were positively correlated (Table 1). All significant correlations in *C. arabica* shade plantations were positive (Table 1A, 1C). In contrast, there were more significant correlations among floral traits in *C. canephora* shade plantations than non-significant ones; and, all but one was positive (Table 1B, 1D). Among the strongest were the correlations between corolla diameter and petal length, and petal length and anther filament length; thus, as one trait in flowers of *C. canephora* sun increased in size, so did most of the others. The number of floral petals affected the allometric relationships of flowers. For example, corolla tube length of *C. canephora* was negatively correlated with petal width for flowers that had 6 petals, but the opposite was true for flowers with 5 petals. There were more significant correlations in the shaded *C. canephora* flowers with 5 petals than 6 (Table 1B, 1D).

**Table 1:** Spearman rank correlation coefficients by species, farm management type (sun vs. shade), and petals (5 or 6 petals) among morphological traits. Bolded values and asterisks indicate significant correlations (*P ≤ 0.05, **P ≤ 0.01, ***P ≤ 0.001). In each sub-table, correlations for two sites are depicted, with one site above the diagonal and another site below the diagonal, as follows: (a) *C. arabica* flowers with 5 petals Shade above and Sun below, (b) *C. canephora* flowers with 5 petals Shade above and Sun below (c) *C. arabica* flowers with 6 petals Shade above and Sun below, (d) *C. canephora* flowers with 6 petals Shade above and Sun below.

		Anther filament	Corolla tube diameter	Corolla diameter	Petal length	Petal width	Style length	Corolla tube length
A	Anther filament	—	0.16*	0.43***	0.26***	0.41	0.24**	0.03
	Corolla tube diameter	0.04	—	0.06	−0.0684	0.41***	0.16*	0.15
	Corolla diameter	0.19	0.01	—	0.78***	0.1	0.19*	0.38***
	Petal length	0.31	0.19	0.71***	—	0	0.19*	0.38***
	Petal width	0.07	0.44*	0.14	0.35*	—	0.13	0.22**
	Style length	0.47**	0.05	0.23	0.33	0.23	—	0
	Corolla tube length	0.17	0.13	0.19	0.31	0.3	0.44*	—
B	Anther filament	—	0.25**	0.30***	0.40***	0.1	0.38***	0.14
	Corolla tube diameter	0.35***	—	0.11	0.13	−0.06	−0.03	0.26**
	Corolla diameter	0.50***	0.26***	—	0.70***	−0.16	0.31***	0.35***
	Petal length	0.52***	0.35***	0.76***	—	−0.25**	0.23**	0.45***
	Petal width	0.16*	0.35***	0.24**	0.22**	—	0.04	−0.23**
	Style length	0.26***	0.16*	0.30***	0.35***	0.09	—	0.21*
	Corolla tube length	0.33***	0.26***	0.49***	0.46***	0.21**	0.24**	—
C	Anther filament	—	0.25	0.33	0.31	−0.34	−0.07	−0.05
	Corolla tube diameter	0.14	—	0.26	0.25	0.25	0.29	−0.29
	Corolla diameter	0.43	0.22	—	0.88***	−0.07	0.27	0.04
	Petal length	0.52	0.3	0.88**	—	0.1	0.31	0.31
	Petal width	0.79*	0.1	−0.05	0.26	—	0.16	0.28
	Style length	0.48	0.4	0.1	0.41	0.67	—	−0.03
	Corolla tube length	0.19	0.49	−0.05	0.21	0.4	0.92***	—
D	Anther filament	—	0.37*	0.26	0.25	0.07	−0.05	0.07
	Corolla tube diameter	0.01	—	0.12	0.16	0.21	−0.023	0.41*
	Corolla diameter	0.39**	0.35*	—	0.75***	0.17	0.35	0.21
	Petal length	0.26	0.30*	0.75***	—	0.18	0.16	0.45*
	Petal width	0.22	0.50***	0.37***	0.14	—	0.24	−0.03
	Style length	0.2	0.14	0.45***	0.39**	0.05	—	−0.18
	Corolla tube length	0.14	−0.1	0.16	0.24	−0.36**	0.11	—

**Figure 2:**
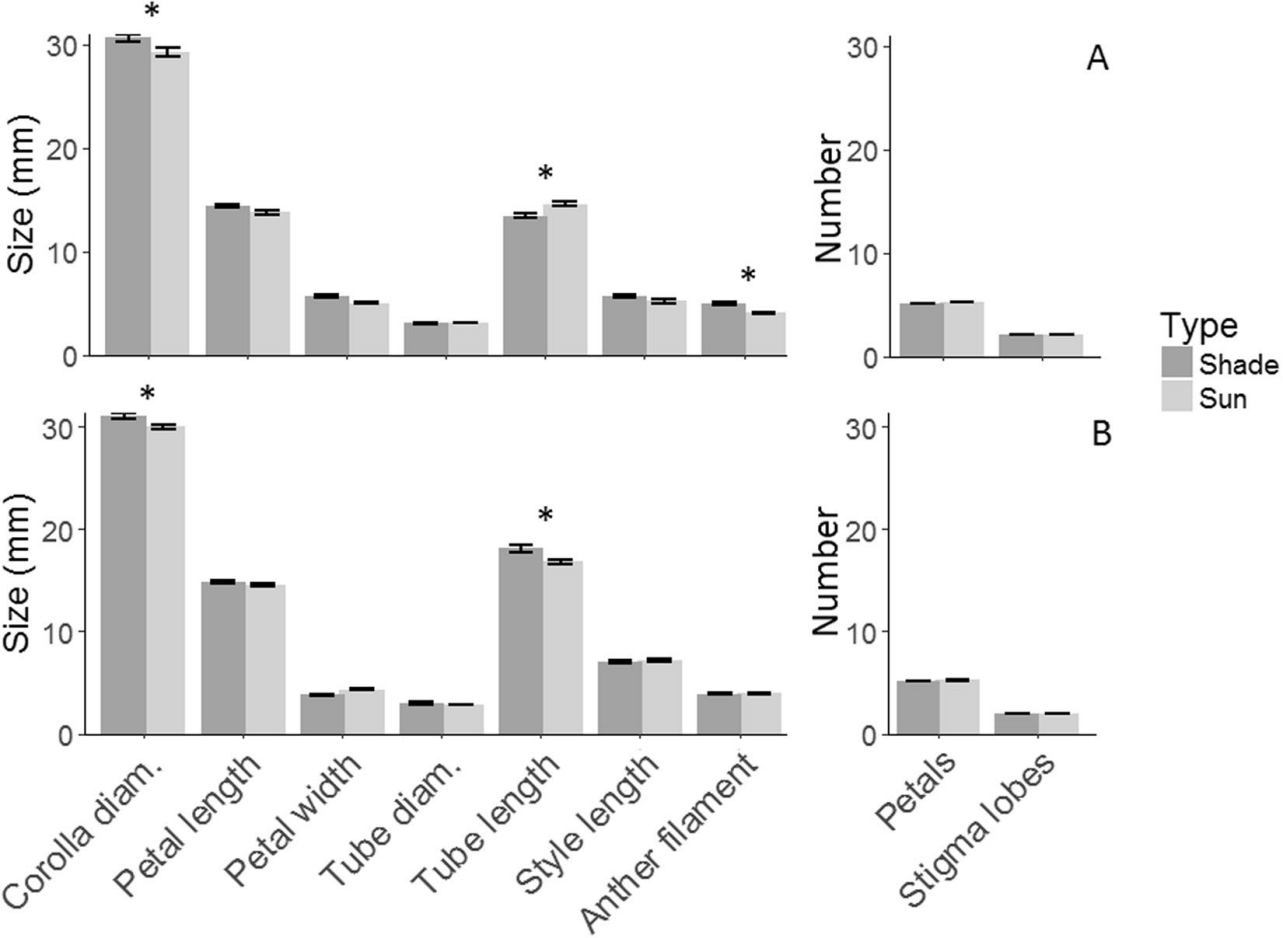
Mean (±SE) floral traits of (**A**) *Coffea arabica* and (**B**) *Coffea canephora*. Asterisks above the bars indicate significant (P < 0.05) differences between means of shade and sun.

Some floral morphological traits differed significantly by species and by farm type. For *C. arabica*, there was only a marginal main effect of farm type on reproductive floral traits (*F*_*1,6*_ = 5.56; *P* = 0.054), a significant main effect of floral trait (*F*_*2,550*_ = 616.86; *P* < 0.001), and a significant interaction between farm type and floral trait (*F*_*2,550*_ = 12.06; *P* < 0.001). Similarly, there was no significant main effect of farm type on floral traits important for visual attraction (*F*_*1,28*_ = 0.4; *P* = 0.53), but there was a significant main effect of floral trait (*F*_*5,13*75_ = 6955.5; *P* < 0.001) and a significant interaction between farm type and floral trait (*F*_*5,1375*_ = 10.5; *P* < 0.001). Specifically, *C. arabica* plants grown under shade exhibited 1.4% larger corolla diameter and 12.8% anther height than when grown in sun (respectively, T_*75*_ = 3; *P* = 0.004; T_12_ = 4.23; *P* = 0.001). Only tube length was significantly larger in sun plantations, being 8.7% larger in sun than shade (T_75_ = −3.22; *P* = 0.002; Fig. 2A).

In contrast, for *C. canephora*, there was no significant main effect of farm type on reproductive floral traits (*F*_*1,9*_ = 0.00; *P* = 0.98), but there was a significant main effect of floral trait (*F*_*2,1189*_ = 1807.19; *P* < 0.001). There was no significant interaction between farm type and floral trait (*F*_*2,1189*_ = 0.16; *P* = 0.85). There was also no significant main effect of farm type on floral traits important for visual attraction (*F*_*1,9*_ = 0.7; *P* = 0.42), but there was a significant main effect of floral trait (*F*_*5,2050*_ = 8835.8; *P* < 0.001) and a significant interaction between farm type and floral trait (*F*_*5,2050*_ = 10.3; *P* < 0.001). Specifically, corolla diameter and tube length were 3.7% and 8.0% larger in shade than sun plantations of *C. canephora* (respectively, T_14_ = −0.14; *P* = 0.03; T_14_ = 2.89; *P* = 0.01; Fig. 2B).

### Nectar standing crop, sugar concentration, and caffeine concentration

**Figure 3:**
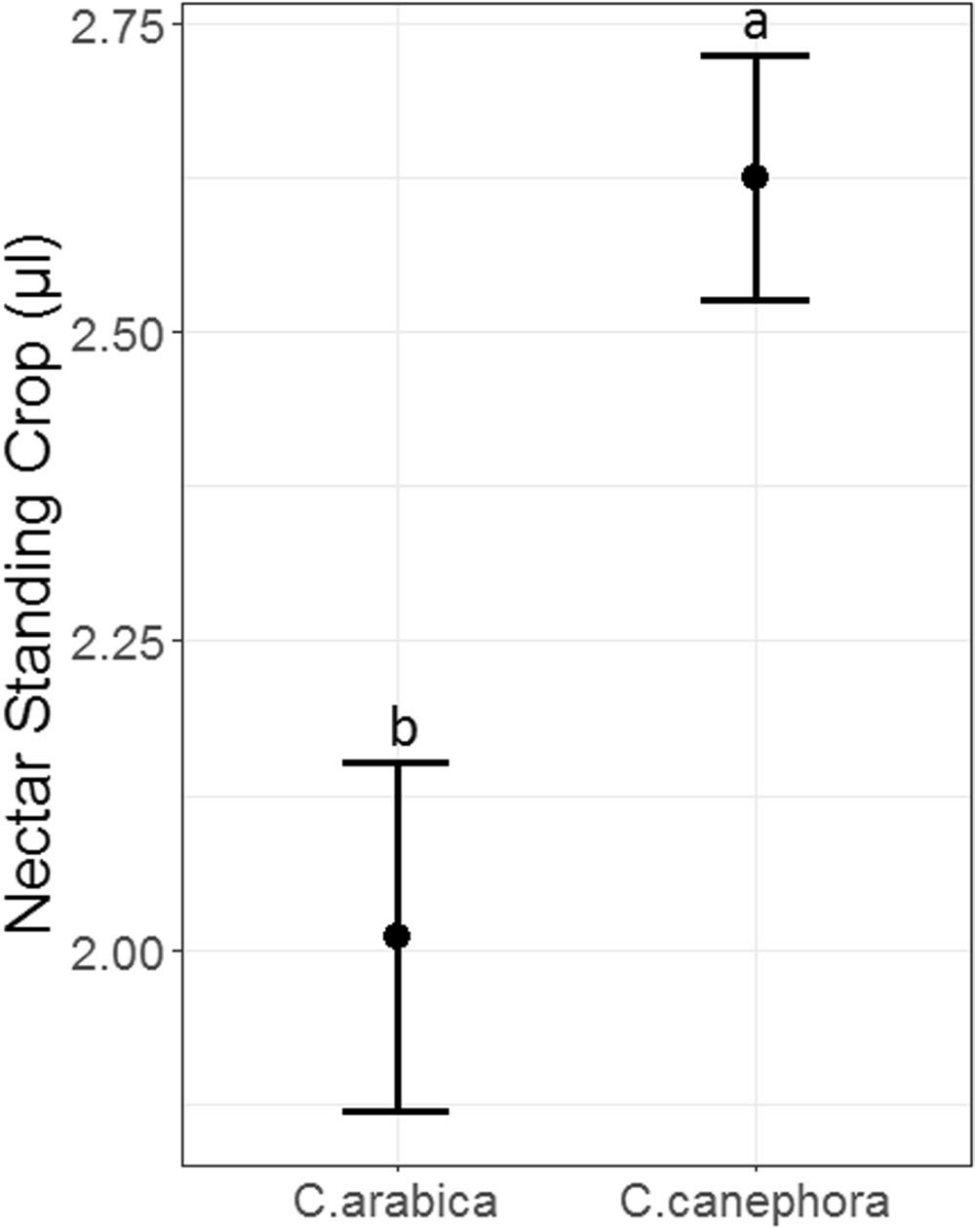
Mean (±SE) nectar volume (µl) from *C. arabica* and *C. canephora* flowers. Different letters indicate a significant (P < 0.05) main effect of species on nectar volume.

**Figure 4:**
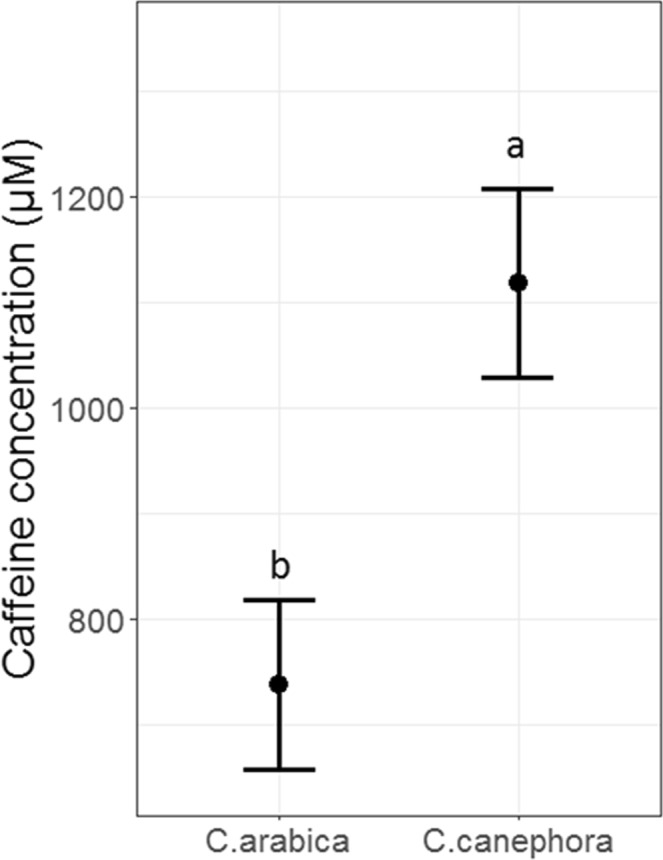
Mean (±SE) nectar caffeine concentration from shade and sun plantations for *C. arabica* and C. canephora. Means with different letters are significantly different at P ≤ 0.05.

Some nectar traits differed significantly between coffee species, but farm management type had no effect on nectar reward traits. Specifically, nectar standing crop differed significantly between species (F_1,70_ = 9.68; P = 0.003), with 1.3-times more nectar in flowers of *C. canephora* than those of *C. arabica* (Fig. 3). Nectar standing crop did not differ by farm type (F_1,49.3_ = 0.0005; P = 0.98), and there was no interaction between species and farm type (F_1,70_ = 0.28; P = 0.60). For nectar sugar concentration, we found no effects of species, farm type, or their interaction (F < 4.04; P > 0.065 in all cases). Across both species and farm types, nectar sugar concentration ranged from 12.6–25.0%. Finally, nectar caffeine concentration was 1.5-times greater for *C. canephora* than *C. arabica* (*F*_*1,11*_ = 11.29; *P* = 0.007; Fig. 4), with no difference in caffeine concentration between farm types (*F*_*1,10*_ = 0.06; *P* = 0.81).

### Pollen production and nitrogen content

**Figure 5:**
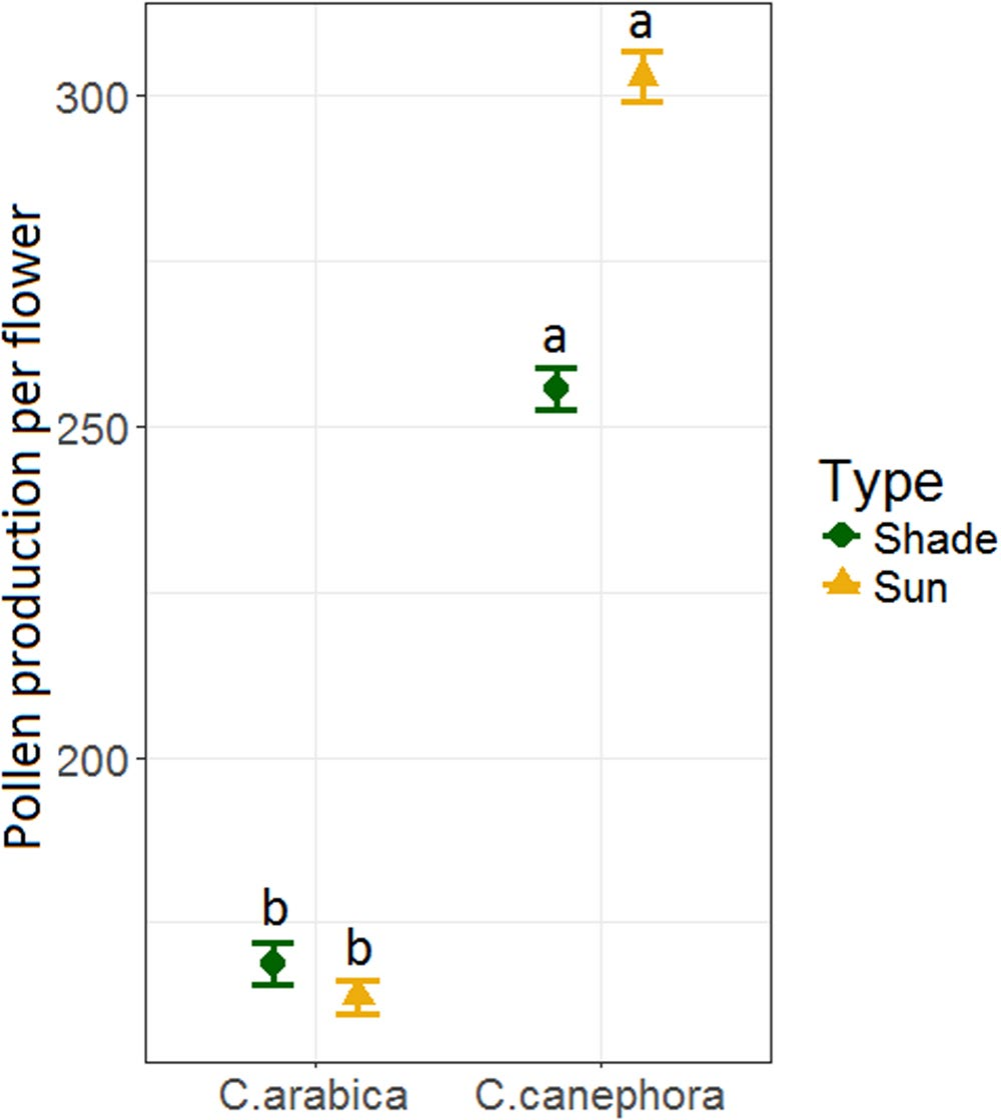
Mean (±SE) pollen production per flower for *C. arabica* and *C. canephora* collected in shade and sun coffee plantations. Means with different letters are significantly different at P < 0.05.

**Figure 6:**
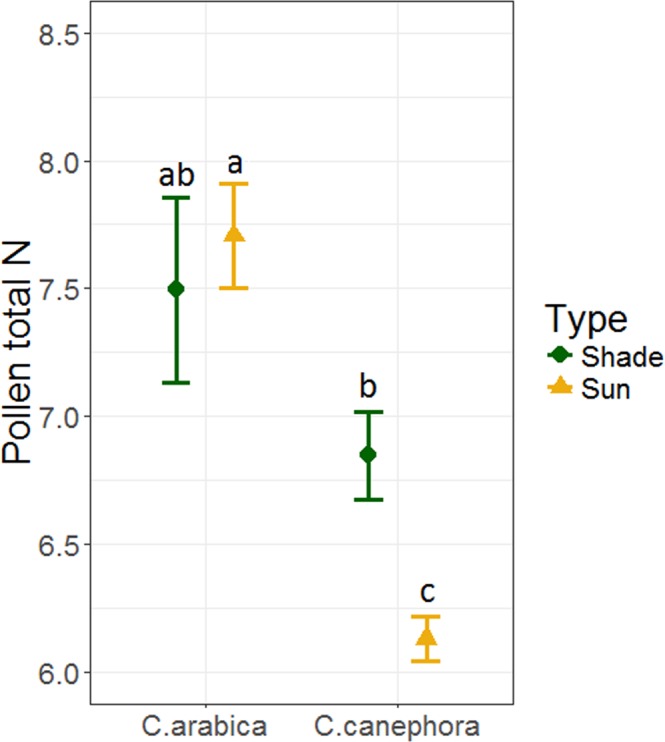
Mean (±SE) pollen total nitrogen (N) content from shade and sun coffee plantations for *C. arabica* and *C. canephora*. Means with different letters are significantly different at P ≤ 0.05.

Pollen production and nitrogen content varied by species and farm management type. For pollen production, we found that *C. canephora* produced 1.7-times more pollen than *C. arabica* (*F*_*1,15*_ = 62.03; *P* < 0.001; Fig. 5). Pollen production did not differ by farm type (F_1,13_ = 0.68; P = 0.43), but there was a marginal effect of the interaction between species and farm type (F_1,15_ = 4.41; P = 0.05). Even so, post-hoc analysis showed no significant difference between pollen production in sun and shade plantations of *C. arabica* or *C. canephora* (T_15_ = 0.98; *P* = 0.76; T_12_ = −2.04; *P* = 0.23). Although *C. canephora* produced more pollen per flower, its pollen had 1.16-times lower total N than *C. arabica* (*F*_*1,36*_ = 33.89; *P* < 0.001; Fig. 6). There was no overall main effect of farm type on pollen N content (*F*_*1,36*_ = 2.11; *P* = 0.16), but there was a significant interaction between species and farm type (*F*_*1,36*_ = 6.40; *P* = 0.02; Fig. 6). Farm type modified pollen N content of the two species differently. For *C. canephora*, pollen from sun farms had significantly lower N content than pollen from shade farms (T_36_ = 3.08; *P* = 0.02). However, for *C. arabica*, there was no significant difference in pollen N content between sun vs. shade (T_36_ = −0.71; *P* = 0.89).

## Discussion

Plants that rely on animal pollinators are dependent on their floral display to attract visitors that can effectively pollinate flowers. We assessed variation in floral morphological, nectar, and pollen traits of two important agricultural species, *Coffea arabica* and *C. canephora*, under different farm management cultivation strategies (sun and shade). Floral traits were generally positively correlated with one another within each species, with a few exceptions. Our results showed that corolla diameter was larger in shade coffee plantations of both *C. arabica* and *C. canephora* and anther filament length was longer in shade plantations of *C. arabica*. Corolla tube length differed in response to shade between both species, with larger tube length in sun for *C. arabica* and shade for *C. canephora*. There was no effect of farm management strategy on nectar standing crop, caffeine concentration, or sugar concentration nor was there an effect on pollen production per flower, but there was a significant difference between species with more nectar, caffeine and pollen per flower being produced in *C. canephora* flowers. Only pollen total nitrogen differed between farm type and species, with more nitrogen found in the pollen of flowers of *C. arabica*, followed by *C. canephora* flowers grown under shade, and then sun. Understanding the ways in which management practices impact floral traits can be especially important for agricultural systems, where variation in these traits could affect variation in pollination and, consequently, yield and profits for pollen-limited systems.

In general, our correlation analyses indicate that many of the floral traits were positively correlated in sun and shade plantations of both species. As such, flowers that are larger in one trait are generally larger overall, and management practices that might have an effect on floral morphological traits will affect these traits in a similar way. Floral traits are often positively correlated with one another in other plant systems^46,47^, suggesting that plants likely exhibit more variation in flower size than flower shape. For example, ccorrelations between related floral morphological traits, and between flower number and plant size in *Erysimum mediohispanicum* (Brassicaceae) have been recorded, but no correlation between corolla shape and any other trait Gomez *et al*.^48^. In our comparison of the effects of management practices on floral traits, we found that three out of the nine floral traits measured differed significantly between sun and shade plantations. Corolla diameter was larger in shade coffee plantations of both *C. arabica* and *C. canephora*, anther filament length was longer in shade plantations of *C. arabica*, and corolla tube length was larger in shade plantations of *C. canephora*. Studies in other floral systems have shown that larger flowers are preferred by bees compared to smaller flowers^49,50^. If this is the case in coffee systems as well, then this would suggest that bees might prefer flowers in shade plantations than sun plantations.

Differences in floral trait size between sun and shade plantations can be due to a variety of abiotic factors, including variation in soil nutrient levels^12,15^, soil moisture^11^, temperature^51^ and incoming solar radiation^14^. For example, high watering regimes resulted in significantly larger calyx lengths, and stigma-anther distance of *Lythrum silicara* compared to medium and low watering regimes^11^. Similarly, *Aquilefia coerulea* plants had longer stigmas in wetter conditions, and shorter anther and stigma lengths in hotter, drier conditions^51^. Given that shade plantations exhibit less microclimatic extremes than sun plantations^21,52^, it is likely that the more constant soil moisture and cooler temperatures^18^ resulted in overall larger floral traits in shade.

It is surprising that although corolla tube length was positively correlated with all floral traits in *C. arabica* sun and the rest of the floral traits were smaller in sun than shade, that corolla tube length was still larger in sun than shade. Corolla tube length can be an important trait influencing pollinator behavior^41,53^. For example, a longer tube relates to a longer distance that must be traversed by the visitor to reach the reward – either with its body or tongue^41^, and this can in turn affect flower handling time. Bumble bees, for instance, handled lavender flowers faster than honey bees, whose tongues were slightly shorter than those of the bumble bees and the average tube length^54^. Unlike many of the floral traits measured, mean corolla tube length was noticeably (1.3 times) larger in *C. canephora* than *C. arabica*, suggesting that longer tongued bees might be more effective at handling *C. canephora* flowers, just as they might be for flowers under shade and sun for *C. canephora* and *C. arabica*, respectively.

Among the floral traits that we considered important for reproduction, anther filament length was the only one that differed between sun and shade plantations, and this difference was only observed in *C. arabica* plants. Anther filament length and style length are important structures for reproduction as they produce and receive pollen. For example, shorter styles and anther filaments, which could be closer to one-another than longer styles and anthers, can result in sexual interference^55^. There are two types of intra-floral interference, one of which involves pollen clogging, whereby self-pollen compromises female function, and the other occurs when the plant parts impede the positioning of the pollinator preventing effective pollination^55^. Differences in the relative sizes of anthers and styles of the two coffee species matched expectations based on their mating systems. Specifically, *C. arabica* is self-compatible, and in this species both style and anther filament lengths were similar (0.85 mm difference), and differed less in shade than sun (0.65 mm vs 1.58 mm, respectively). This similarity in anther and style lengths may result in autogamous self-pollen transfer and pollination insurance in cases where flowers do not receive outcrossed pollen. Nonetheless, *C. arabica* fruit production has been shown to benefit from cross-pollination^56^, so there may be some detrimental effects on pollination in shaded plants if sexual interference is occurring. There was a much larger difference in size of these reproductively important traits in *C. canephora* (3.2 mm), the self-incompatible species, that relies on cross-pollination for effective fruit set. In this case, the spatial separation may reduce self-pollen deposition from anthers to stigmas, but experiments are needed to test this hypothesis.

Nectar sugar concentration surprisingly did not differ across the type of farm or species. These results differ from those of Wright *et al*.^23^ who found that *C. arabica* had a higher sugar concentration than *C. canephora*. Field measurements of nectar sugar concentration can be influenced by temperature and humidity^57,58^. Thus, it is possible that differences in environmental conditions between the two management practices drove differences in the nectar sugar concentration results (SG Prado, *unpubl. res*.). Alternatively, rainfall may have played a role in balancing out nectar sugar concentrations in both treatments, as many of the flowers we sampled experienced afternoon or early morning rainfall prior to sampling. Although we made sure to collect nectar samples from flowers that were angled sideways or downward, we cannot rule out the possibility that they received some rainwater. Increased volume, viscosity and sugar concentrations in nectar have all been shown to increase bee handling times^57,59^ and handling time has in turn been linked to greater pollen transfer by bees^59^. As such, the self-incompatible *C. canephora* plants may be benefiting from improved pollination services compared to the self-compatible *C. arabica* plants.

The nectar of *Coffea* flowers not only contained sugars but also the alkaloid caffeine. Consistent with Wright *et al*.^23^, we observed a higher caffeine concentration in *C. canephora* than *C. arabica* flowers; however, for both species, the caffeine concentrations were much higher (ca. 4 and 30 times greater for *C. canephora* and *C. arabica*, respectively). Previous studies have suggested that caffeine may have a stronger effect on bee olfactory memory than sugar concentration, resulting in bees becoming more likely to prefer and return to plants with those similar caffeinated signals^23,24^. However, the caffeine concentration of *C. canephora* flowers in both shade and sun farms in our study exceeded prior studies, with our flowers containing mean caffeine concentrations above 1000 µM. Such concentrations have been shown to have the opposite effect on bees, diminishing a bee’s ability to learn and may be a deterrent to honey bees^30^. As such, the likelihood that the caffeine in *C. canephora* is ensuring pollinator fidelity might be lower than for *C. arabica*. This would suggest that bee pollination of *C. canephora* might be compromised, potentially making it more dependent on abiotic pollination for seed set^60^.

Pollen production per flower did not differ between sun and shade plantations but did differ between species. Flowers of *C. canephora* had significantly more pollen per flower than those of *C. arabica*. As pollen is the male gamete of the plant, there’s a trade-off experienced by the plant to maximize reproduction, while also attracting and rewarding flower visitors^1^. Therefore, producing more pollen may be one way the plant ensures sufficient pollen transferred for reproduction^61^. This would be especially important for *C. canephora* as it relies on animal and wind pollination for fruit set, and thus not all pollen grains produced will successfully reach conspecifics. Alternatively, *C. canephora* might simply require greater pollen deposition than *C. arabica* for successful fruit set^62^. Contrary to pollen production, pollen total N content was greater in *C. arabica* pollen than *C. canephora*, and greater in shade plantations of *C. canephora* than sun. Pollen N content has been shown to vary between species of many flowering plants, including *Hibiscus* spp. and *Passiflora* spp.^62^ and such variation between species might explain our observed differences between *C. arabica* and *C. canephora*. Similarly, to nectar, plant pollen characteristics can differ with environmental factors, and therefore differences in environmental conditions may help explain these results. For example, high levels of phosphorus in soils of *Cucurbita pepo* can result in pollen that also contains higher concentrations of phosphorus^63,64^. It is therefore possible that the differences in nitrogen content are due to the different levels of nitrogen found in sun and shade plantations (e.g., nitrogen-fixing leguminous trees, slow release through leaf litter decomposition in shade and chemical fertilizers in sun). Additional research is needed to identify the ways in which different nitrogen inputs and nitrogen release times affect pollen protein content.

The comparative work conducted in this study is a necessary first step in understanding the relationship between large-scale agricultural practices and changes in floral traits. We found that corolla diameter, corolla tube length and pollen total nitrogen content were greater in shade plantations of *C. canephora* than sun plantations. Likewise, corolla tube length and anther filament length were larger in shade plantations of *C. arabica*. As larger floral displays are generally preferred by bees^49,50^ and higher nitrogen content results in increased net nutritional gains, the variation in floral traits in shade plantations might benefit plant pollination and pollinators alike. This study not only helps fill a gap in knowledge about the effects of shade trees on floral traits, which can be pertinent to other agroforestry systems, but to our knowledge, it is also the first study to provide baseline data on *C. arabica* and *C. canephora* floral traits. As such, it lays a foundation upon which to formulate hypotheses to investigate causal mechanisms underlying pollinator-coffee relationships.

## Methods

### Study system

#### Study area

This study was conducted from January 2017 through April 2017 at 16 coffee plantations located in the central and western part of Puerto Rico (Table 2). The 16 farms varied in size (0.393–31.44 ha) and agricultural practices (Table S1). All of the *C. canephora* farms used also had *C. arabica* planted. Two of the farms were used for both *C. canephora* and *C. arabica* floral trait measurements. Four farms were in sun and five under shade for *C. arabica*, and five in sun and four under shade for *C. canephora*. All of the farms had coffee rust (*Hemileia vastatrix*), although *C. canpehora* plants were less affected than *C. arabica* plants. Five focal coffee plants per species were selected randomly within each of the farms, and all floral trait measurements were taken from these same plants. When possible, *C. arabica* var. Bourbon was sampled. All *C. canephora* were of the same variety - Robusta.

**Table 2:** Latitude and longitude of the 16 coffee farms studied.

Species	Type	Latitude	Longitude
*C. arabica*	Sun	18.14587	−66.9003
	Sun	18.15235	−66.9297
	Sun	18.14956	−66.8909
	Sun	18.15443	−66.9349
	Shade	18.26836	−66.6105
	Shade	18.26667	−66.6118
	Shade	18.26339	−66.6164
*C. canephora*	Sun	18.21347	−66.7924
	Sun	18.21846	−67.004
	Sun	18.22101	−67.0034
	Sun	18.21149	−66.7943
	Sun	18.1994	−66.7831
	Shade	18.18637	−66.8121
	Shade	18.18637	−66.8121
*C. canephora* & *C. arabica*	Shade	18.26959	−66.6119
	Shade	18.2617	−66.6161

The land-cover in these regions is classified as lowland moist and montane wet evergreen coffee plantations^65^. Elevations in these regions ranged from 375–875 m.a.s.l., with mean annual rainfall between 1743–2428 mm and mean annual temperatures between 21.6–25.7 °C^66^. In Puerto Rico, there are two rainy seasons, a short one in April–May and a long one in September–December. Likewise, there are two dry seasons, a short one between June–August and a long one between January–March.

Coffee cultivation in Puerto Rico experienced a period of nearly 20 years of agricultural intensification^67^, starting in the late 1980s, resulting in a drastic increase in the number of sun coffee farms^68^. It is only recently that specialized shade coffee (plantations with a restored shade layer; Fig. S1) have been adopted as an alternative to strike a better balance between conservation and coffee production. These two cultivation practices (sun vs. shade) create contrasting environmental conditions, some of which are directly attributable to management practices. For example, sun coffee plantations rely less on ecological processes than shade plantations, replacing them with various agrochemicals, including fertilizers, insecticides and herbicides^69^. Moreover, the excessive use of these agrochemicals can contribute to high levels of soil erosion^70^ and nutrient leaching^16^. In contrast, restoring the shade layer can convey some resilience to increasing daytime temperatures, maintain a moister and cooler microsphere than sun coffee plantations, and provide a buffer against extreme climate events, such as hurricanes^18,71^. These conditions can help improve plant growth and development by maintaining or improving soil fertility directly by reducing erosion^18^ or indirectly through the addition of leaf litter^69^ and nitrogen fixation, in the case of leguminous shade trees^16^. Conversely, there are physiological drawbacks, such as resource competition, when shade trees are planted within coffee plantations^18^. Shade vs. sun cultivation may therefore have different effects on floral traits.

#### Study species

Both *Coffea arabica* and *C. canephora* are native to the African equatorial forest^72^. *Coffea arabica*, which is native to the Ethiopian tropical forests, can be cultivated between a range of 800–2000 m, and *C. canephora*, which is native to the lowland forests of the Congo river basin can be grown between <500–1500 m^42,72^. Optimal rainfall for *C. arabica* ranges between 1200–1800 mm, and temperatures between 18–21 °C^42^. *Coffea canephora* in turn, can adapt to intensive rainfalls exceeding 2000 mm and has an optimal mean temperature ranging between 22–30 °C^42^. Unlike *C. arabica*, *C. canephora* thrives under high air humidity^42^. *Coffea canephora* is self-incompatible and *C. arabica* is self-compatible, although it has been shown to experience increased yield from cross-pollination by bees^56^. Green beans of *C. canephora* contain more caffeine and have a higher concentration of caffeine than those of *C. arabica* (2.2% vs. 1.2% of dry mass, respectively)^44,45^. Similarly, leaves of *C. canephora* also contain more caffeine than those of *C. arabica* (3% vs. 1.6% of dry weight, respectively^73^).

In Costa Rica and Mexico, the main pollinators of coffee were found to be social bees in the genera *Melipona* and *Trigona* as well as *Apis mellifera*^74,75^. In Puerto Rico, an island with over 35 species of bees, the main pollinator seen in coffee plantations was *A. mellifera* (SGP, personal observations), the only social bee on the island^76^. A *Lasioglossum* species and *Xylocopa mordax* were also observed pollinating the coffee flowers, but these sightings were rare (SGP, personal observations).

### Floral shape

To study the morphological variation of *C. canephora* and *C. arabica* flowers, for each species we randomly selected ten open flowers on the five focal bushes within each farm. We collected measurements in all but two farms, resulting in a sample of 66 bushes. A total of 729 flowers were measured, 369 of which were of *C. canephora* (207 sun, 162 shade), and 360 of which were of *C. arabica* (180 sun, 180 shade). To describe floral traits important for visual attraction of pollinators, we measured the following on each flower: petal width and length, corolla diameter, corolla tube length, corolla tube diameter at opening, and counted the number of petals (Fig. 1). To describe variation in reproductive traits that can affect the ability of insects to pollinate^3,55^, we measured anther filament length, style length, and number of stigmatic lobes (Fig. 1). Measurements were taken using a Mitutoyo digital calliper to the nearest 0.01 mm (Model No. 500-196-30, Mitutoyo, Auroral, Illinois, USA).

### Floral nectar sugar concentration and standing crop

A total of 67 nectar sugar concentration readings were taken, 47 for *C. canephora* (38 sun, 9 shade), and 20 for *C. arabica* (12 sun, 8 shade). A total of 249 nectar standing crop measurements were taken, with 160 taken from *C. canephora* (130 sun, 30 shade) and 89 from *C. arabica* (50 sun, 39 shade). To measure nectar standing crop per flower, we bagged several bunches of flowers which were 1–2 days from blooming, using bridal veil fabric, to exclude floral visitors. Once the flowers bloomed, we removed the fabric, and collected nectar from 10 randomly selected flowers. We sampled nectar using 5 and 10 µL microcapillary tubes inserted into the base of the flower; we did not squeeze flowers for nectar collection but instead allowed the nectar to suck into the tubes via capillary action. Samples were taken between 9:00–14:00, during which time temperatures ranged from 23–32 °C and windspeeds ranged between 0 and 4.7 Km/h. To measure total sugar concentration, we collected approx. 20 µl of nectar from one or more flowers, as necessary, and measured concentration on an Atago 2352 Master-53T hand-held refractometer with automatic temperature compensation (Atago, Bellevue, Washington, USA), and noted the sugar concentration to the nearest 0.5%. Nectar from the standing crop measurements was used, and if more nectar was necessary to obtain the 20 µl for the sample, then nectar was extracted from additional flowers on the same coffee plant.

### Floral nectar caffeine content

Using 5–54 flowers from the same coffee plants, we collected 43 nectar samples of between 20–35 µl to measure nectar caffeine content (*C. arabica*: 8 shade, 10 sun; *C. canephora*: 13 shade, 12 sun). We immediately placed the nectar samples into a cooler with ice. They were then stored in a freezer at 0 **°**C until they were lyophilized. Each sample was then diluted with 100 µl of methanol. Samples (5 µl) were analyzed directly by liquid chromatography-mass spectroscopy using a Dionex UltiMate 3000 LC system with separation of compounds on a Phenomenex Luna C18(2) column (150 Å~3 mm i.d., 3 μm particle size) at 400 μL min^−1^ and eluted using a linear gradient of 90:0:10 (t = 0 min) to 0:90:10 (t = 20–25 min), returning to 90:0:10 (t = 27–30 min). Solvents were water, methanol and 1% formic acid in acetonitrile, respectively. The column was maintained at 30 °C. Compounds were detected by MS on a Thermo Fisher Velos Pro Dual-Pressure Linear Ion Trap Mass Spectrometer. Samples were scanned, using FTMS, from m/z 194–196 corresponding to the molecular ion for caffeine (M + H = *m/z* 195.1) in positive mode. Peak areas were quantified against a calibration curve of an authentic caffeine standard (Sigma, Dorset, UK).

### Pollen production and nitrogen content

Using 1–10 flowers per coffee plant, we collected anthers from a total of 11 plants in 4 *C. arabica* shade plantations, 12 plants in 4 *C. arabica* sun plantations, 14 plants in 4 *C. canephora* shade plantations, and 10 plants in 2 *C. canephora* sun plantations. A total of 481 flowers were used to measure pollen production per flower (*C. arabica* – 96 shade, 120 sun; *C. canephora* – 126 shade, 139 sun). To measure pollen production per flower, we bagged several bunches of flowers which were 1–2 days from blooming, using bridal veil fabric, to exclude floral visitors. Once the flowers bloomed, we removed the fabric, and collected the anthers from 10 randomly selected flowers, placing the anthers from each flower into separate microcentrifuge tubes. To remove the pollen from the anthers, we added 1500 µl of 70% ethanol to each microcentrifuge tube and sonicated the tubes for 5 minutes to release the pollen from the anther sacs. We then vortexed the samples for approximately 10 seconds, moving the pollen into suspension in the tube. We extracted 4 µl of the suspended solution and placed it on a hemocytometer and counted the number of coffee pollen grains under a dissecting microscope (Nikon SMZ1000) at 20X magnification. We counted 6 subsamples from each tube. We then took the mean of the subsamples and used that mean to calculate the number of pollen grains in the original 1500 µl of liquid (hereafter pollen grains per flower).

We also used some of the freshly opened, bagged flowers, to collect pollen for nitrogen (N) analysis. We removed 12–18 randomly selected flowers from 39 of our focal bushes, and using an electric toothbrush, we vibrated the flower, with the anthers placed within a microcentrifuge tube, to release pollen from the anther sacs. Pollen samples were kept in a freezer at 0 °C until processing. We added 400 µl of 200-proof ethanol to each tube and centrifuged on low RPM for 15 seconds to move the pollen to the bottom of the tube. We removed excess ethanol with a pipette and allowed any remaining ethanol to evaporate off over 24 hr. Pollen samples were then stored in the freezer at −30 °C until analysis. The 39 samples were sent to the UC Davis Analytical Laboratory (Davis, CA, USA) to determine total N using combustion with a LECO FP-528 and TruSpec CN Analyzers. Total N can be used as a proxy for crude total protein content in pollen^77^. Three of the 39 samples had an insufficient amount of pollen for analysis, leaving 36 samples for statistical analysis. Pollen for the 36 samples came from 5 plants in 3 shade *C. arabica* plantations, 11 plants in 4 sun *C. arabica* plantations, and 8 plants in 2 sun *C. canephora* plantations, 12 plants in 4 shade *C. canephora* plantations.

### Data analysis

All statistical analyses were performed in R studio (Version 1.0.44). We used Spearman’s rank nonparametric correlation analyses to assess the degree to which *Coffea* floral traits were related to one another using package Biotools and Hmisc^78,79^. Data were grouped by farm management types (sun/shade), species within management type, and the number of petals (5 or 6) within species. The allometric relationships of floral traits were evaluated within the context of farm management types (sun/shade), species within management type, and the number of petals (5 or 6) within species. To assess variability in floral shape of each coffee species further, we grouped floral traits into two categories: those important for attracting pollinators (petal width and length, corolla diameter, corolla tube length, corolla tube diameter at opening, and the number of petals) and those important for reproduction (anther filament length, style length, and number of stigmatic lobes). We tested whether these traits differed between sun and shade plantations of *C. arabica* and *C. canephora* using four linear mixed effect models (LMER)– one for each category of floral traits. In these models, fixed effects were: farm type (sun vs. shade) and traits measured; and random effects were flower nested within bush nested within farm. Although we conducted multiple tests, we followed the guidelines of Moran^80^ and Gotelli and Ellison^81^ and report unadjusted P-values.

We used a LMER to compare nectar standing crop, sugar concentration, and caffeine concentration between species and shade and sun plantations. We square-root transformed nectar standing crop and caffeine concentration to improve normality. One value for caffeine concentration was removed from analysis as it was an outlier, being 7 times greater than any of the other concentrations found for *C. arabica*. We also used a LMER to compare pollen production per flower (square-root transformed) and total pollen N (log-transformed) between sun and shade coffee plantations. In the models for nectar sugar concentration, nectar standing crop and pollen production per flower, fixed effects included species (*C. canephora* and *C. arabica*) and farm type (sun vs. shade), and random effects included flower nested within bush, and bush nested within farm. For nectar caffeine concentration and pollen total N, we used a similar model but only included bush nested within farm as the random effect. A post-hoc test was performed for caffeine concentration, pollen production per flower, and pollen total N, given that there were two-way interactions between coffee species and farm type. We used package lmerTest for the LMER analyses, and lsmeans for the post-hoc analyses^82,83^.

## Supplementary information

Supplementary Table S1 and Figure S1

## References

[1] Willmer, P. G. *Pollination and Floral Ecology*. (Princeton University Press, 2011).

[2] Klein A-M (2007). Importance of pollinators in changing landscapes for world crops. Proc. Biol. Sci..

[3] Nishihiro J, Washitani I, Thomson JD, Thomson BA (2000). Patterns and consequences of stigma height variation in a natural population of a distylous plant, Primula sieboldii. Funct. Ecol..

[4] Stone JL, Thomson JD (1994). The evolution of distyly: pollen transfer in artificial flowers. Evolution (N. Y)..

[5] Parachnowitsch AL, Kessler A (2010). Pollinators exert natural selection on flower size and floral display in Penstemon digitalis. New Phytol..

[6] Cariveau D (2004). Direct and indirect effects of pollinators and seed predators to selection on plant and floral traits. Oikos.

[7] Carruthers JM (2017). Oilseed rape (Brassica napus) as a resource for farmland insect pollinators: quantifying floral traits in conventional varieties and breeding systems. GCB Bioenergy.

[8] Egan PA (2018). Crop Domestication Alters Floral Reward Chemistry With Potential Consequences for Pollinator Health. Front. Plant Sci..

[9] López-Uribe, M. M., Cane, J. H., Minckley, R. L. & Danforth, B. N. Crop domestication facilitated rapid geographical expansion of a specialist pollinator, the squash bee Peponapis pruinosa. *Proc. R. Soc. B Biol. Sci*. **283** (2016). 10.1098/rspb.2016.0443 PMC4936030 27335417

[10] Brock MT, Weinig C (2007). Plasticity and environment-specific covariances: An investigation of floral-vegetative and within flower correlations. Evolution (N. Y)..

[11] Mal TK, Lovett-Doust J (2005). Phenotypic plasticity in vegetative and reproductive traits in an invasive weed, Lythrum salicaria (Lythraceae), in response to soil moisture. Am. J. Bot..

[12] Burkle LA, Irwin RE (2010). Beyond biomass: measuring the effects of community-level nitrogen enrichment on floral traits, pollinator visitation and plant reproduction. J. Ecol..

[13] Petanidou T, Smets E (1996). Does temperature stress induce nectar secretion in Mediterranean plants?. New Phytol..

[14] Kilkenny FF, Galloway LF (2008). Reproductive success in varying light environments: Direct and indirect effects of light on plants and pollinators. Oecologia.

[15] Hoover SER (2012). Warming, CO_2_, and nitrogen deposition interactively affect a plant-pollinator mutualism. Ecol. Lett..

[16] Beer J (1988). Litter production and nutrient cycling in coffee (*Coffea arabica*) or cacao (Theobroma cacao) plantations with shade trees. Agrofor. Syst..

[17] Szott LT, Kass DCL (1993). Fertilizers in agroforestry systems. Agrofor. Syst..

[18] Beer J, Muschler R, Kass D, Somarriba E (1998). Shade management in coffee and cacao plantations. Agrofor. Syst..

[19] DaMatta FM, Ronchi CP, Maestri M, Barros RS (2007). Ecophysiology of coffee growth and production. Brazilian J. Plant Physiol..

[20] Damatta FM, Ramalho JDC (2006). Impacts of drought and temperature stress on coffee physiology and production: a review. Brazilian J. Plant Physiol..

[21] Lin BB (2007). Agroforestry management as an adaptive strategy against potential microclimate extremes in coffee agriculture. Agric. For. Meteorol..

[22] Thomson James D., Draguleasa Miruna A., Tan Marcus Guorui (2015). Flowers with caffeinated nectar receive more pollination. Arthropod-Plant Interactions.

[23] Wright GA (2013). Caffeine in floral nectar enhances a pollinator’s memory of reward. Science.

[24] Singaravelan N, Ne’eman G, Inbar M, Izhaki I (2005). Feeding responses of free-flying honeybees to secondary compounds mimicking floral nectars. J. Chem. Ecol..

[25] Strauss SY, Conner JK, Rush SL (1997). Foliar Herbivory Affects Floral Characters and Plant Attractiveness to Pollinators: Implications for Male and Female Plant Fitness. Am. Nat..

[26] Jones PL, Agrawal AA (2016). Consequences of toxic secondary compounds in nectar for mutualist bees and antagonist butterflies. Ecology.

[27] Stevenson PC, Nicolson SW, Wright GA (2017). Plant secondary metabolites in nectar: impacts on pollinators and ecological functions. Funct. Ecol..

[28] Arnold SEJ, Idrovo MEP, Arias LJL, Belmain SR, Stevenson PC (2014). Herbivore Defence Compounds Occur in Pollen and Reduce Bumblebee Colony Fitness. J. Chem. Ecol..

[29] Tiedeken EJ (2016). Nectar chemistry modulates the impact of an invasive plant on native pollinators. Funct. Ecol..

[30] Mustard JA, Dews L, Brugato A, Dey K, Wright GA (2012). Consumption of an acute dose of caffeine reduces acquisition but not memory in the honey bee. Behav. Brain Res..

[31] Barlow SE (2017). Distasteful Nectar Deters Floral Robbery. Curr. Biol..

[32] Irwin RE, Cook D, Richardson LL, Manson JS, Gardner DR (2014). Secondary compounds in floral rewards of toxic rangeland plants: Impacts on pollinators. J. Agric. Food Chem..

[33] Richardson LL, Bowers MD, Irwin RE (2016). Nectar chemistry mediates the behavior of parasitized bees: Consequences for plant fitness. Ecology.

[34] Vaast P, Bertrand B, Perriot J-J, Guyot B, Génard M (2006). Fruit thinning and shade improve bean characteristics and beverage quality of coffee (*Coffea arabica* L.) under optimal conditions. J. Sci. Food Agric..

[35] Irwin RE, Adler LS (2006). Correlations among traits associated with herbivore resistance and pollination: implications for pollination and nectar robbing in a distylous plant. Am. J. Bot..

[36] Adler LS, Wink M, Distl M, Lentz AJ (2006). Leaf herbivory and nutrients increase nectar alkaloids. Ecol. Lett..

[37] Cenicafé. In *Manual del cafetero colombiano: Investigacion y tecnologia para la sostenibilidad de la caficultura* (eds Gast, H. F. *et al*.) 117- (LEGIS, 2013).

[38] Lim T. K. (2012). Coffea arabica. Edible Medicinal And Non-Medicinal Plants.

[39] Lim T. K. (2012). Coffea canephora. Edible Medicinal And Non-Medicinal Plants.

[40] Dyer AG (2012). Parallel evolution of angiosperm colour signals: common evolutionary pressures linked to hymenopteran vision. Proc. R. Soc. B Biol. Sci..

[41] Dafni A, Lehrer M, Kevan PG (1997). Spatial flower parameters and insect spatial vision. Biol. Rev. Camb. Philos. Soc..

[42] DaMatta FM (2004). Ecophysiological constraints on the production of shaded and unshaded coffee: a review. F. Crop. Res..

[43] Worley AC, Barrett SCH (2000). Evolution of floral display in Eichhornia paniculata (Pontederiaceae): direct and correlated responses to selection on flower size and number. Evolution (N. Y)..

[44] Elements, T. & Raton, B. *Green Coffee*. 1481–1487 (2003).

[45] Clarke, R. J. In *Encyclopedia of Food Sciences and Nutrition* 51, 1481–1487 (Elsevier, 2003).

[46] Young H, Stanton M, Ellstrand N, Clegg J (1994). Temporal and spatial variation in heritability and genetic correlations among floral traits in Raphanus sativus, wild radish. Heredity (Edinb)..

[47] Davis SL (2001). Phenotypic and genetic correlations among floral traits in two species of Thalictrum. J. Hered..

[48] Gómez JM, Torices R, Lorite J, Klingenberg CP, Perfectti F (2016). The role of pollinators in the evolution of corolla shape variation, disparity and integration in a highly diversified plant family with a conserved floral bauplan. Ann. Bot..

[49] Makino TT, Sakai S (2007). Experience changes pollinator responses to floral display size: From size-based to reward-based foraging. Funct. Ecol..

[50] Stanton ML, Preston RE (1988). Ecological Consequences and Phenotypic Correlates of Petal Size Variation in Wild Radish, Raphanus sativus (Brassicaceae) Author (s): Maureen L. Stanton and Robert E. Preston Published by: Botanical Society of America, Inc. Stable URL: http://w. Am. J. Bot..

[51] Van Etten ML, Brunet J (2013). The Impact of Global Warming on Floral Traits That Affect the Selfing Rate in a High-Altitude Plant. Int. J. Plant Sci..

[52] Staver C, Guharay F, Monterroso D, Muschler RG (2001). Designing pest-suppressive multistrata perennial crop systems: shade-grown coffee in Central America. Agrofor. Syst..

[53] Galen C, Cuba J (2001). Down the tube: Pollinators, predators, and the evolution of flower shape in the alpine skypilot, Polemonium viscosum. Evolution (N. Y)..

[54] Balfour NJ, Garbuzov M, Ratnieks FLW (2013). Longer tongues and swifter handling: Why do more bumble bees (Bombus spp.) than honey bees (Apis mellifera) forage on lavender (Lavandula spp.)?. Ecol. Entomol..

[55] Barrett SCH (2002). Sexual interference of the floral kind. Heredity (Edinb)..

[56] Roubik DW (2002). The value of bees to the coffee harvest. Nature.

[57] Roubik DW, Buchmann SL (1984). Nectar selection by Melipona and Apis mellifera (Hymenoptera: Apidae) and the ecology of nectar intake by bee colonies in a tropical forest. Oecologia.

[58] Corbet SA, Unwin DM, Prŷs-Jones OE (1979). Humidity, nectar and insect visits to flowers, with special reference to Crataegus, Tilia and Echium. Ecol. Entomol..

[59] Thomson JD (1986). Pollen Transport and Deposition by Bumble Bees in Erythronium: Influences of Floral Nectar and Bee Grooming. J. Ecol..

[60] Willmer PG, Stone GN (1989). Incidence of entomophilous pollination of lowland coffee (*Coffea canephora*); the rold of leaf cutter bees in Papua New Guinea. Entomol. Exp. Appl..

[61] Burd M (1994). Bateman’s principle and plant reproduction: The role of pollen limitation in fruit and seed set. Bot. Rev..

[62] Roulston TH, Cane JH, Buchmann SL (2000). What Governs Protein Content of Pollen: Pollinator Preferences, Pollen–Pistil Interactions, or Phylogeny?. Ecol. Monogr..

[63] Lau T-C, Stephenson AG (1993). Effects of Soil Nitrogen on Pollen Production, Pollen Grain Size, and Pollen Performance in Cucurbita pepo (Cucurbitaceae). Am. J. Bot..

[64] Lau TC, Stephenson AG (1994). Effects of Soil-Phosphorus on Pollen Production, Pollen Size, Pollen Phosphorus-Content, and the Ability to Sire Seeds in Cucurbita-Pepo (Cucurbitaceae). Sex. Plant Reprod..

[65] Parés-Ramos, I. K., Gould, W. A. & Aide, T. M. Agricultural abandonment, suburban growth, and forest expansion in Puerto Rico between 1991 and 2000. *Ecol. Soc*. **13** (2008).

[66] NOAA. National Weather Service Forecast Office. (2018).

[67] Vilsack, T. & Clark, C. Z. F. *Puerto Rico Island and Municipio Data. 2007 Census of agriculture* **1** (2009).

[68] Borkhataria R, Collazo JA, Groom MJ, Jordan-Garcia A (2012). Shade-grown coffee in Puerto Rico: Opportunities to preserve biodiversity while reinvigorating a struggling agricultural commodity. Agric. Ecosyst. Environ..

[69] Haggar J (2011). Coffee agroecosystem performance under full sun, shade, conventional and organic management regimes in Central America. Agrofor. Syst..

[70] Danse, M. & Wolters, T. Sustainable coffee in the mainstream: The case of the SUSCOF Consortium in Costa Rica. *Greener Manag. Int*. 37–51 (2003).

[71] Schroth G (2009). Towards a climate change adaptation strategy for coffee communities and ecosystems in the Sierra Madre de Chiapas, Mexico. Mitig. Adapt. Strateg. Glob. Chang..

[72] Herrera Juan Carlos, Lambot Charles (2017). The Coffee Tree—Genetic Diversity and Origin. The Craft and Science of Coffee.

[73] Perrois C (2014). Differential regulation of caffeine metabolism in *Coffea arabica* (Arabica) and *Coffea canephora* (Robusta). Planta.

[74] Ricketts TH (2004). Tropical Forest Fragments Enhance Pollinator Activity in Nearby Coffee Crops. Conserv. Biol..

[75] Vergara CH, Badano EI (2009). Pollinator diversity increases fruit production in Mexican coffee plantations: The importance of rustic management systems. Agric. Ecosyst. Environ..

[76] Genaro, J. A. & Franz, N. M. The bees of Greater Puerto Rico (Hymenoptera: Apoidea: Anthophila). *Insecta mundi* 1–24 (2008).

[77] Milton, K. & Dintzis, F. R. Nitrogen-to-Protein Conversion Factors for Tropical Plant Samples Author (s): Katharine Milton and Frederick R. Dintzis Published by: Association for Tropical Biology and Conservation Stable, http://www.jstor.org/stable/2388122 Accessed: 27-07-2. Assoc. *Trop. Biol. Conserv*. **13**, 177–181 (1981).

[78] Harrell, F. E. Jr. Hmisc: Harrell Miscellaneous. (2018).

[79] da Silva, A. R. biotools: Tools for Biometry and Applied Statistics in Agricultural Science (2017).

[80] Moran MD (2003). Arguments for rejecting the sequentional bonferroni in ecological studies. Oikos.

[81] Gotelli, N. J. & Ellison, A. M. *A primer of ecological statistics*. (Sinauer, 2004).

[82] Searle S. R., Speed F. M., Milliken G. A. (1980). Population Marginal Means in the Linear Model: An Alternative to Least Squares Means. The American Statistician.

[83] Kuznetsova, A., Brockhoff, P. B. & Christensen, R. H. B. lmerTest: Tests in Linear Mixed Effects Models. (2016).

