# Supplementary material for: A comparison of coffee floral traits under two different agricultural practices
Source: Sci Rep. 2019 May 14;9:7331. doi: 10.1038/s41598-019-43753-y (PMC6517588; doi:10.1038/s41598-019-43753-y)
Supplement: Supplementary file 1 — Supplementary Table S1 and Figure S1 [file 41598_2019_43753_MOESM1_ESM.docx]

Title**: A comparison of coffee floral traits under two different agricultural practices**

Authors: Sara Guiti Prado, Jaime A. Collazo, Philip C. Stevenson and Rebecca E. Irwin

| **Species** | **Type** | **Cultivated land (acres)** | **Coffee age (years)** | **Variety** | **yield 2013 (100 Kg)** | **yield 2014 (100 Kg)** | **yield 2015 (100 Kg)** | **Non-coffee crop** | **Frequency of pesticide application** | **Frequency of herbicide application** | **Frequency of fertilizer application** | **Method for weed control** |
| --- | --- | --- | --- | --- | --- | --- | --- | --- | --- | --- | --- | --- |
| Arabica | Shade | 5.83 | 7 | limani, caturra | NA | 23 | 10 | plantain | 1-2/year | 3-4/year | 2-3/year | herbicide |
| Arabica | Shade | 2.43 | 15-18 | catimorra, paca | NA | 7 | 6.5 | plantain and orange | none | 1-2/year | 1/year | machete |
| Arabica | Sun | 38.84 | 10 | pais, mundo nuevo, limani, fronton | 80 | 55 | 55 | banana, plantain, orange, mandarine, lemon | 1/year | 2-3/year | 2-3/year | machete and herbicide |
| Arabica | Sun | 67.97 | NA | caturra, limani, fronton | 200-300 | 89 | NA | plantain, banana, orange | 2/year | none | 1/year | machete and herbicide |
| Arabica | Sun | 12.62 | 2 to 5 | limani, catuay | NA | NA | 30 | orange, mandarine | none | 3/year | 2/year | herbicide |
| Arabica | Sun | 77.68 | 10 to 12 | caturra, bourbon, fronton, limani, catuay | NA | NA | NA | orange | 2/year | 6/year | 2/year | herbicide |
| Arabica | Sun | 67.97 | 7 | caturra, bourbon, fronton, limani, catuay | NA | NA | NA | orange | 2/year | 6/year | 2/year | herbicide |
| Arabica and Canephora | Shade | 4.86 | 20 | limani, caturra | 50 | 40 | 15 | banana and plantain | 2/year | 2-3/year | none | machete and herbicide |
| Arabica and Canephora | Shade | 9.71 | 8 to 11 | fronton, limani | NA | NA | 14 | mandarine | 5-6/year | 4/year | 4/year | machete and herbicide |
| Canephora | Shade | 3.88 | 20 | robusta | NA | NA | 7 | orange, mandarine, plantain, grapefruit, yautia root, pinneapple, banana | none | 2-3/year | 2/year | machete and herbicide |
| Canephora | Shade | 3.88 | 15-20 | robusta | NA | NA | 15 | plantain and orange | none | 3/year | 2/year | machete and herbicide |
| Canephora | Shade | 29.13 | 8 | robusta | NA | 60-75 | 70-75 | banana, orange, lemons, cacao | NA | NA | NA | machete |
| Canephora | Sun | 4.86 | 1 | robusta | NA | NA | NA | plantain | 2-3/year | 3/year | 3/year | machete and herbicide |
| Canephora | Sun | 21.36 | NA | robusta | NA | NA | NA | orange, mandarine, soursop, sugar apple, sapote and avocado | 1/year | all year as needed | 3/year | herbicide |
| Canephora | Sun | 4.86 | 20 | robusta | NA | NA | 125 | orange, mandarine, plantain | all year as needed | 2/year | 2/year | machete and herbicide |
| Canephora | Sun | 30.59 | 2 to 5 | robusta | NA | NA | 100 | orange, plantain, avocado, mango, lemon | 2/year | all year as needed | 2/year | saw, machete and herbicide |

**Table S1.** Data detailing agricultural practices obtained from oral interviews carried out with the farmers in 2016.


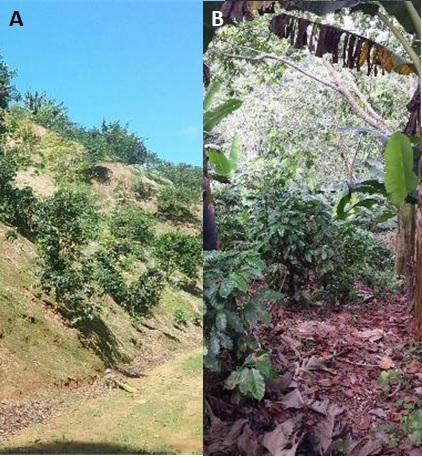


**Figure S1**. Photo of a (A) Sun and (B) Specialized shade coffee plantation.
